# Cases in Practice

**Published:** 1856-02

**Authors:** Benj. Woodward

**Affiliations:** Sharon, Ill.


ARTICLE II.—*Cases in Practice.* By BENJ. WOODWARD, M. D., Sharon, Ill.

CASE I.—The following case was at the time, one of deep interest to me; and thinking it might be so to the profession at large—offer it for the pages of the *Journal.*

On the 27th of January last, my little girl, Florence, was cracking and eating some Hickory nuts, and having some in her mouth, laughed at something and got a piece of shell (as was subsequently proved) in the trachæ. She coughed convulsively for a few moments and then appeared relieved. The next day in the afternoon she had a chill, followed by fever, with every symptom of pneumonia. For this I treated her. There was nothing in the sound of her cough (which was slight) or the breathing peculiar, but the stethescope revealed a whistling sound low down in the right bronchia. I did not at this time suspect the presence of any foreign body in the lungs, but looked upon the pneumonia as caused by the irritation of coughing the day before. Mercurials, antimonials, ipecac, digitalis, cuping blisters, opiates, all the ordinary means were tried, but in vain, the disease went on. On the 8th day from the attack, the pulse was 160 per minute, small and like a fine cord vibrating under the finger.

On the 9th day, she was attacked with all the symptoms of pleurisy on the right side. Blisters were renewed and the severity of the symptoms somewhat abated, but there was no abatement of the fever. On the 10th day, she had a severe chill, and from this time to the end of the 27th day, there were chills with hectic fever every afternoon. Quinine and the nitro muriatic acid were resorted to, but without avail. She seemed to sink gradually till the morning of the 31st day, when she was taken coughing violently, and very soon a stream of pure pus issued from her mouth, which continued for some time, when she sunk into apparent death. In about an hour she was taken coughing again, when pus was again discharged, and with it a piece of hickory nut shell, weighing when dry a little more than two grains. It is rather more then one-fourth of an inch long, and one-eighth wide.

She had not swallowed a mouthful of nourishment from the 10th day till after the abcess broke, but we kept up her strength by enemata of strong animal broths, with nitro muriatic acid and quinine. From the 20th to the 31st day, she lay like one dead, neither shaking or moving, and her breath hardly perceptible. Through the whole of her sickness after the 10th day, neither percussion or the stethescope revealed any sign of vitality in the right lobe of the lung. About the 15th or 16th day, the heart's action became impaired, sometimes every 5th, 6th, and 7th pulsation would be wanting, and as the disease advanced the intermissions would be still more frequent. All this time there was no enlargement of the right side of the thorax or swelling in the intercostal spaces, but on the 33d day, the breathing became still more difficult, and I thought, I could distinguish fluctuation just to the right of the bottom of the sternum, and on plunging in a trochar a stream of pus issued which continued to discharge for some days. From this time her improvement was constant, but slow. Before her sickness she was a fleshy, healthy, child, but since that time she has been frail and delicate.

At no time, up to the present, has there been any motion of the right side, nor will the stethescope reveal any sound.

The above case, (aside from its being my own child), has for me peculiar interest. I have thus made a plain statement of the facts in the case, and will neither weary you nor your readers with any theories.

